# The Successful Treatment of Primary and Recurrent Ruptured Gastric Gastrointestinal Stromal Tumor (GIST) Through Coordinated Surgical Intervention and c-Kit Inhibitor Therapy

**DOI:** 10.7759/cureus.100880

**Published:** 2026-01-05

**Authors:** Shintaro Hirayama, Yujo Kawashita, Masaki Tateishi, Takashi Ueda, Junzo Yamaguchi, Masashi Haraguchi, Kouya Umeda, Masayuki Nakamura, Sousei Abe, Seiko Harada, Yasuo Washida, Yoichi Hachitanda

**Affiliations:** 1 Surgery, Fukuoka Seishukai Hospital, Fukuoka, JPN; 2 Radiology, Fukuoka Seishukai Hospital, Fukuoka, JPN; 3 Pathology, Fukuoka Seishukai Hospital, Fukuoka, JPN

**Keywords:** gastric gist, gastrointestinal stromal tumor (gist), imatinib mesylate, neoplasm recurrence, tumor rupture

## Abstract

Gastrointestinal stromal tumors (GISTs) are the most common mesenchymal tumors of the gastrointestinal tract, with the stomach as the most frequent site of occurrence. Spontaneous rupture of gastric GISTs is rare but represents a life-threatening complication requiring emergency intervention. We report a case of a 29-year-old male who presented with the sudden onset of severe abdominal pain following the spontaneous rupture of a giant gastric GIST. Emergency laparotomy and tumor resection were performed, with histopathological examination confirming a high-risk GIST with positive c-Kit (CD117) and CD34 immunostaining. The patient subsequently received imatinib mesylate as adjuvant therapy but temporarily discontinued treatment due to family planning concerns. Three years after discontinuation of imatinib, he developed tumor recurrence, which responded favorably to reintroduction of the c-Kit inhibitor. This report highlights the importance of timely surgical management of ruptured GISTs, the efficacy of imatinib in both adjuvant and recurrence settings, and the need to consider treatment interruption for reproductive planning. We also review the literature on c-Kit inhibitor therapy in GIST management and discuss emerging evidence on imatinib’s effects on fertility, as well as potential strategies for balancing oncological and reproductive goals in young patients with high-risk GIST.

## Introduction

Gastrointestinal stromal tumors (GISTs) account for approximately 1-3% of all gastrointestinal malignancies, with an estimated annual incidence of 10-15 cases per million individuals [1]. Despite their rarity, GISTs represent the most common mesenchymal tumors of the gastrointestinal tract, most frequently arising in the stomach (60-70%), followed by the small intestine and other gastrointestinal sites [2]. Most GISTs harbor activating mutations in the c-KIT proto-oncogene, leading to constitutive activation of receptor tyrosine kinase signaling pathways [2,3]. This molecular feature has enabled the use of tyrosine kinase inhibitors (TKIs), particularly imatinib mesylate, which has revolutionized the treatment of GISTs in both adjuvant and advanced disease settings [4].

Tumor rupture is an uncommon but critical complication of GISTs, occurring in approximately 3-8% of cases, and is associated with peritoneal dissemination and an extremely high risk of recurrence [5,6]. Tumor rupture is now recognized as an independent adverse prognostic factor, often warranting prolonged or intensified adjuvant therapy [7]. However, the optimal management of ruptured GISTs remains challenging, especially in young patients who may face long-term treatment-related life planning issues.

## Case presentation

**Figure 1:**
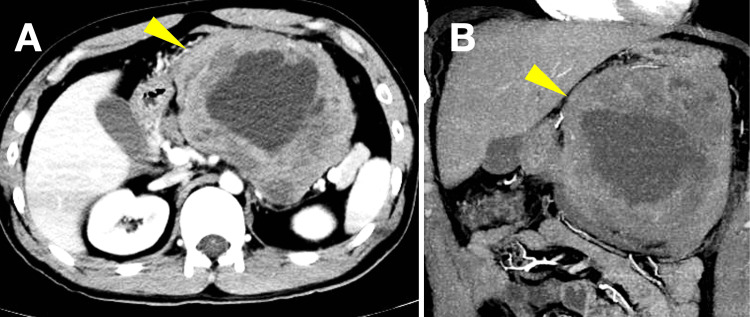
Preoperative contrast-enhanced CT (A) Axial view demonstrating a large heterogeneous mass (arrowhead) arising from the stomach with internal hemorrhage and areas of necrosis. (B) Coronal view showing the extent of the tumor (arrowhead) and associated hemoperitoneum with free fluid in the peritoneal cavity CT: computed tomography

A 29-year-old man with no significant past medical history presented to the emergency department with sudden-onset severe upper abdominal pain that had begun while playing golf. He denied nausea, vomiting, or prior similar episodes. His social history included smoking approximately 20 cigarettes per day for nine years and daily alcohol use. There was no relevant family history. On physical examination, the patient appeared acutely distressed with abdominal distension and marked epigastric tenderness. Laboratory testing revealed leukocytosis (12,030/μL) with normal hemoglobin and platelet counts and mildly elevated C-reactive protein (Table 1). Contrast-enhanced CT scan demonstrated a large heterogeneous mass arising from the stomach with internal hemorrhage and free intraperitoneal fluid, findings consistent with spontaneous tumor rupture and hemoperitoneum (Figure 1).

**Table 1:** Laboratory findings at initial presentation TP: total protein; Alb: albumin; T-Bil: total bilirubin; AST: aspartate aminotransferase; ALT: alanine aminotransferase; γ-GTP: gamma-glutamyl transpeptidase; ALP: alkaline phosphatase; T-cho: total cholesterol; TG: triglycerides; BUN: blood urea nitrogen; Cre: creatinine; WBC: white blood cell count; %Neut: percentage of neutrophils; %Lymph: percentage of lymphocytes; %Mono: percentage of monocytes; %Eosino: percentage of eosinophils; %Baso: percentage of basophils; RBC: red blood cell count; Hb: hemoglobin; Ht: hematocrit; Plt: platelet count; CEA: carcinoembryonic antigen; CA19-9: carbohydrate antigen 19-9; PT: prothrombin time; PT-INR: prothrombin time-international normalized ratio

Laboratory parameters	Values	Normal range
TP	7.0 g/dL	6.7-8.3 g/dL
Alb	4.3 g/dL	4.0-5.0 g/dL
T-Bil	0.50 mg/dL	0.30-1.20 mg/dL
AST	18 U/L	13-33 U/L
ALT	26 U/L	6-30 U/L
γ-GTP	51 U/L	10-47 U/L
ALP	303 U/L	38-113 U/L
T-cho	147 mg/dL	120-219 mg/dL
TG	102 mg/dL	30-149 mg/dL
BUN	10.3 mg/dL	8.0-22.0 mg/dL
Cre	0.65 mg/dL	0.60-1.10 mg/dL
Sodium	139 mEq/L	138-146 mEq/L
Potassium	4.4 mEq/L	3.6-4.9 mEq/L
Chloride	107 mEq/L	99-109 mEq/L
WBC	12030 /μL	3,300-9,000 /μL
%Neut	88.2%	
%Lymph	7.1%	
%Mono	2.4%	
%Eosio	1.8%	
%Baso	0.2%	
RBC	502 ×10⁴/μL	430-570 ×10⁴/μL
Hb	15.0 g/dL	13.5-17.5 g/dL
Ht	45.0%	42.0-53.0%
Plt	3.8 ×10⁴/μL	1.20-3.50 ×10^4^/μL
CEA	0.9 ng/mL	0-5.0 ng/mL
CA19-9	3.6 U/mL	0-37.0 U/mL
PT	102.7 %	70.0-100.0%
PT-INR	0.98	0.85-1.15

**Figure 2:**
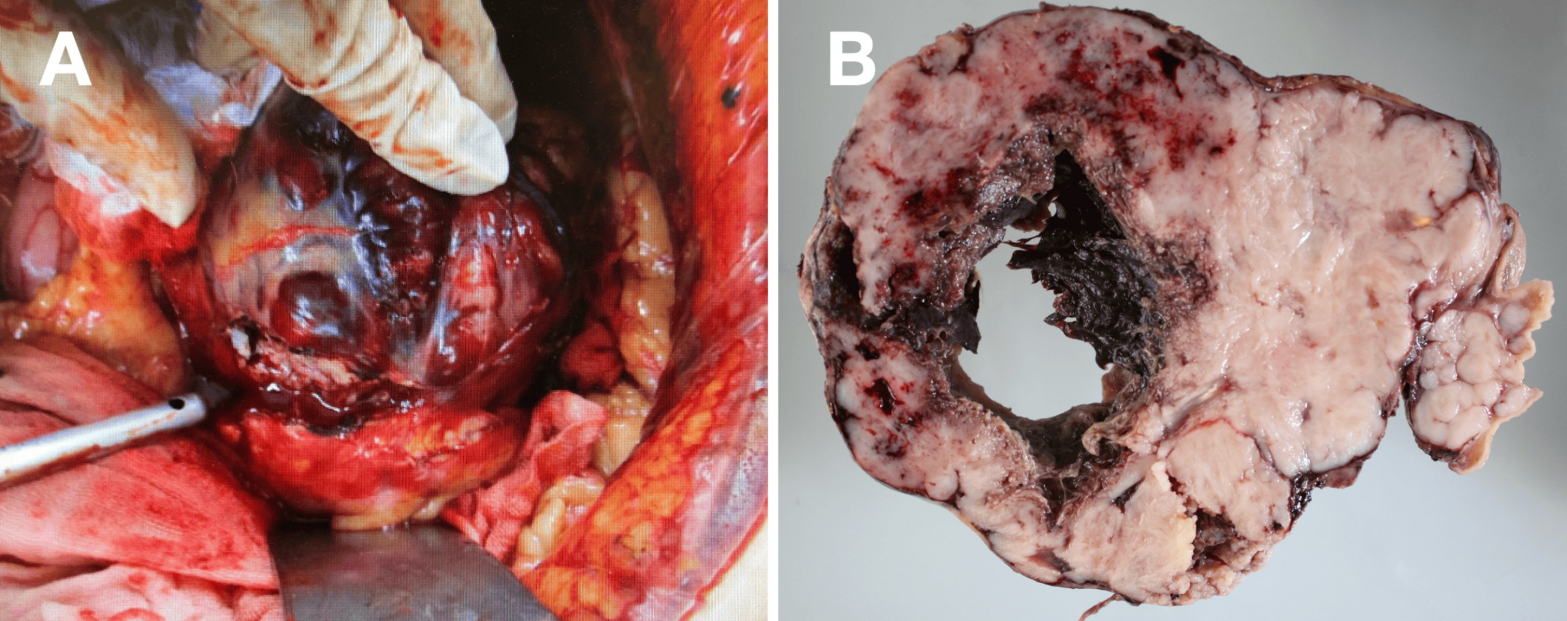
Intraoperative and gross pathological findings (A) Intraoperative view showing the ruptured hemorrhagic gastric tumor with active bleeding. (B) Resected specimen revealing extensive hemorrhage and central necrosis. Operative time: 170 minutes; blood loss: 280 mL (no transfusion)

Emergency exploratory laparotomy revealed a ruptured, hemorrhagic gastric tumor measuring approximately 15 cm in diameter, with active bleeding and accumulation of blood within the peritoneal cavity. Partial gastrectomy with complete tumor resection was performed. The operative time was 170 minutes, and the estimated blood loss was 280 mL, with no transfusion required (Figure 2).

**Figure 3:**
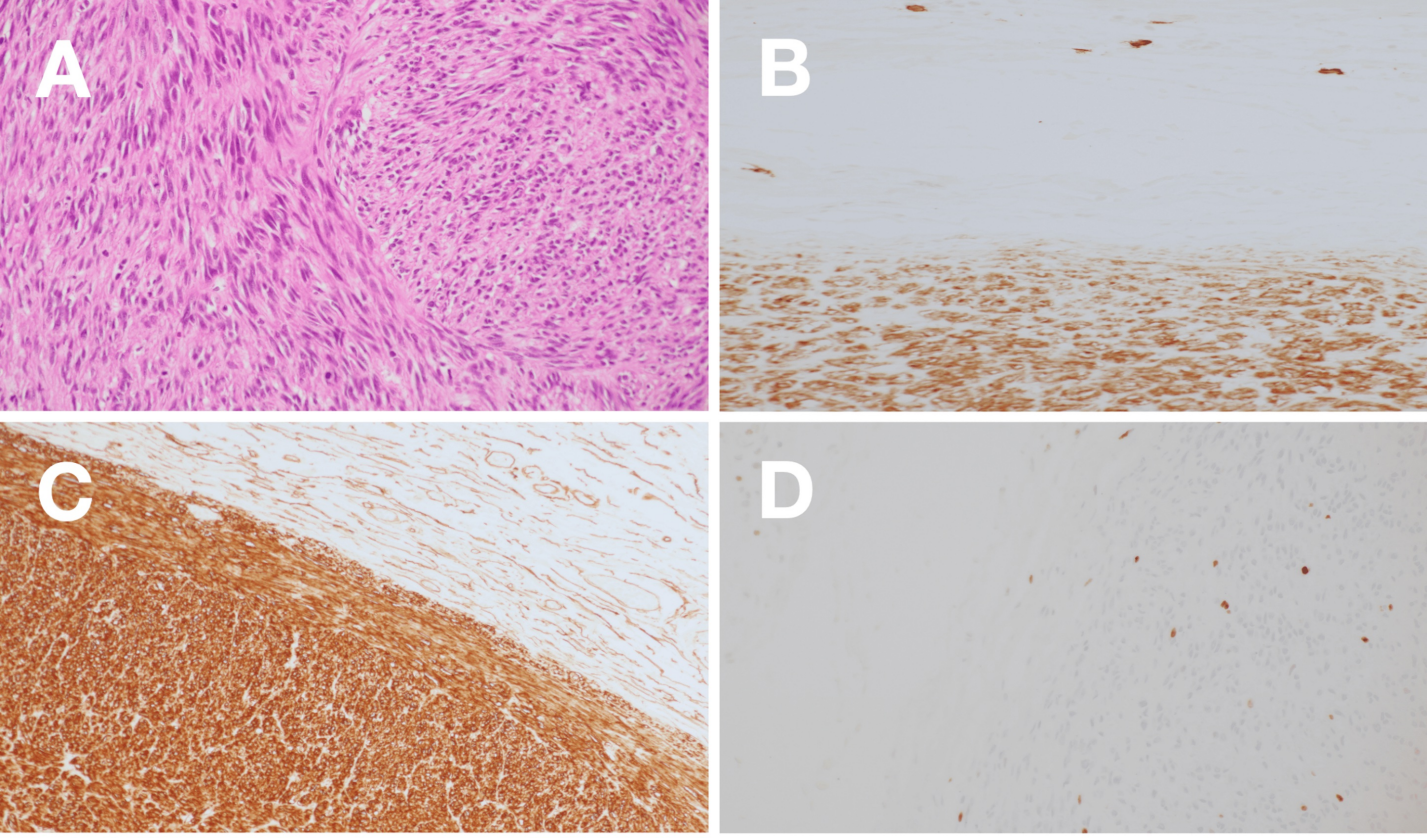
Histopathological examination of the primary tumor (A) Hematoxylin and eosin staining showing spindle cell morphology with fascicular growth pattern. Immunohistochemical staining demonstrating positivity for (B) CD34, (C) KIT (CD117), and (D) Ki-67 (approximately 5% proliferation index)

Gross pathological examination revealed a well-circumscribed tumor with extensive hemorrhage and necrosis. Histopathological analysis showed a spindle cell neoplasm with a fascicular growth pattern. Immunohistochemical staining demonstrated strong positivity for KIT (CD117) and CD34, with a Ki-67 proliferation index of approximately 5%, confirming the diagnosis of a high-risk gastric GIST (Figure 3).

Based on tumor size greater than 10 cm, mitotic activity of 4 per 50 high-power fields, and tumor rupture, adjuvant imatinib therapy at a dose of 400 mg/day was initiated. After two years of adjuvant imatinib therapy, the patient expressed a desire to start a family. Following multidisciplinary discussion and counseling regarding oncologic risks, imatinib was temporarily discontinued. During the treatment-free interval, the patient married and successfully fathered two children.

**Figure 4:**
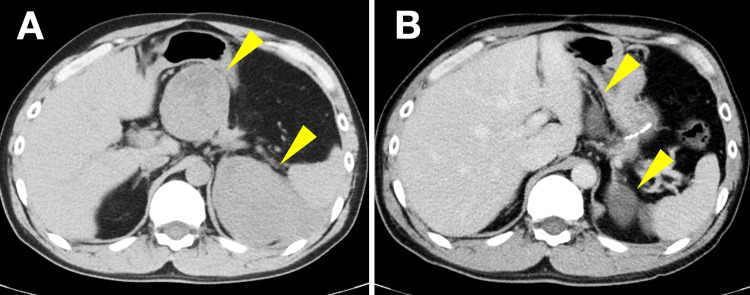
Surveillance CT demonstrating recurrent disease and treatment response (A) Image three years after imatinib discontinuation showing two recurrent lesions (arrowheads): 34 mm adjacent to the pancreatic body and 47 mm lateral to the spleen (sum: 81 mm). (B) Image after imatinib reintroduction demonstrating partial response per RECIST 1.1 criteria with lesion reduction to 14 mm and 18 mm (sum: 32 mm; 60.5% decrease) CT: computed tomography

Three years after discontinuation of imatinib, surveillance CT revealed two recurrent lesions, one measuring 34 mm adjacent to the pancreatic body and another measuring 47 mm lateral to the spleen (Figure 4A). Imatinib therapy was reinitiated at 400 mg/day. Follow-up CT imaging demonstrated a reduction in lesion size to 14 mm and 18 mm, representing a 60.5% decrease in the sum of target lesion diameters from 81 mm to 32 mm and meeting RECIST 1.1 criteria for partial response (Figure 4B).

**Figure 5:**
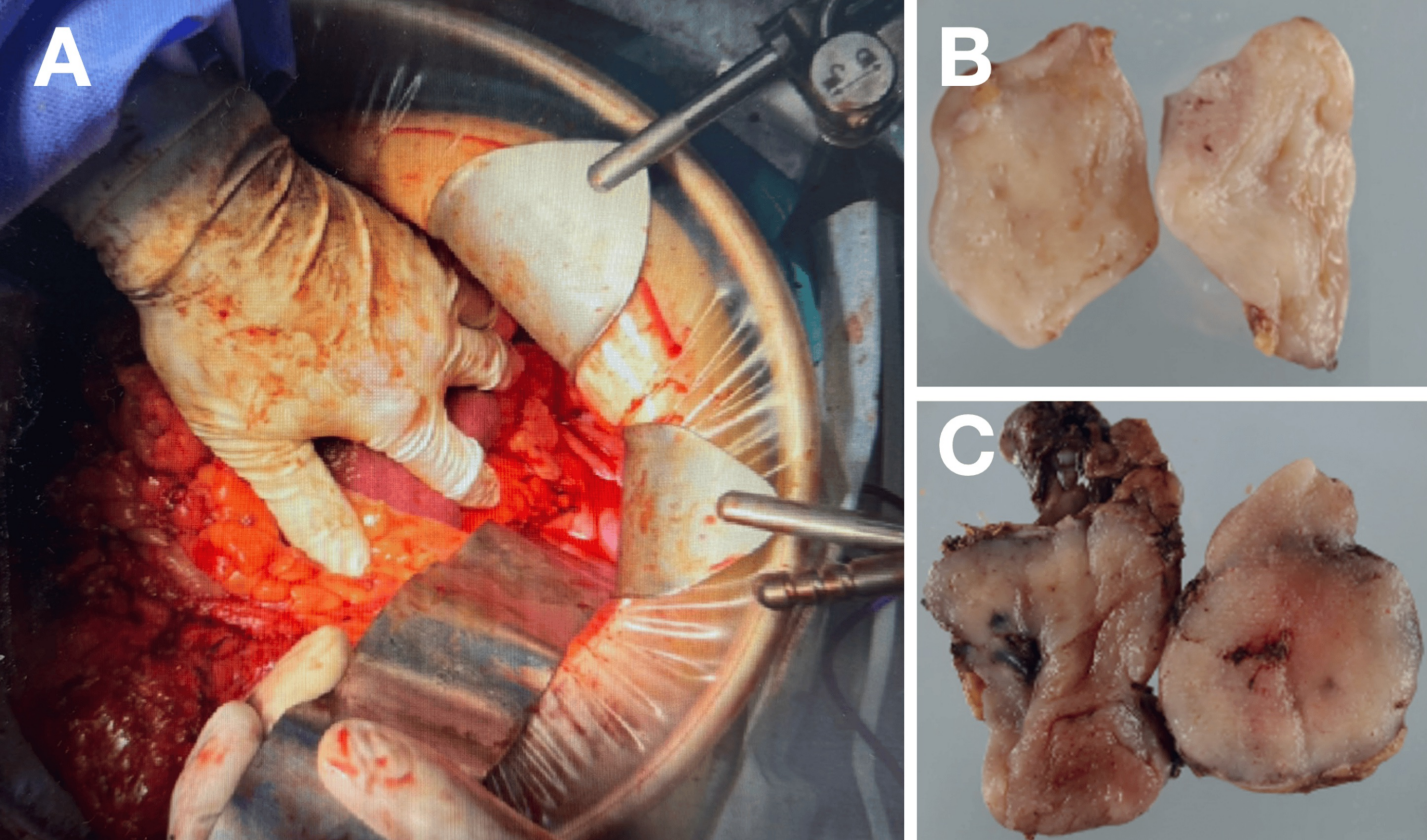
Repeat surgical resection findings Intraoperative view (left) and resected specimens (right) showing recurrent tumors from the superior pancreatic body region (upper right) and lateral splenic region (lower right)

After confirmation of tumor response, the patient underwent surgical resection with complete excision of both lesions (Figure 5).

**Figure 6:**
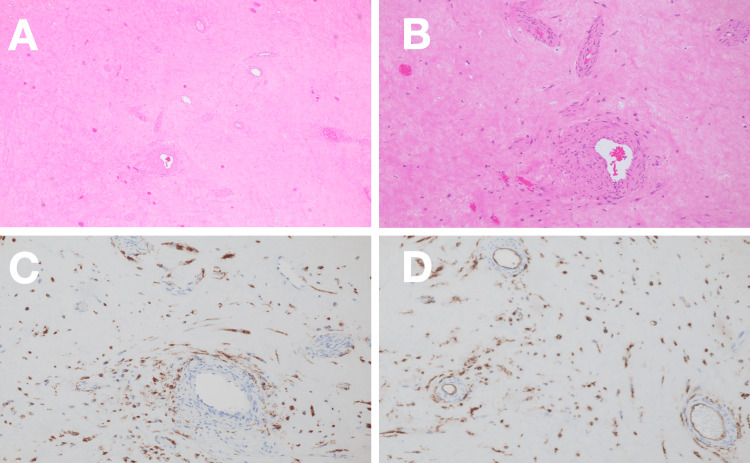
Histopathological examination of recurrent tumors (A, B) Hematoxylin and eosin staining demonstrating marked stromal fibrosis with scattered residual tumor cells, a finding consistent with imatinib-induced tumor regression and suggesting preserved drug sensitivity. (C, D) Immunohistochemical staining showing persistent KIT (CD117) positivity in residual tumor cells, supporting continued eligibility for c-Kit inhibitor therapy

Histopathological findings of the recurrent tumors were consistent with GIST, showing similar morphology to the primary tumor with increased stromal fibrosis and persistent KIT and CD34 expression (Figure 6).

**Figure 7:**
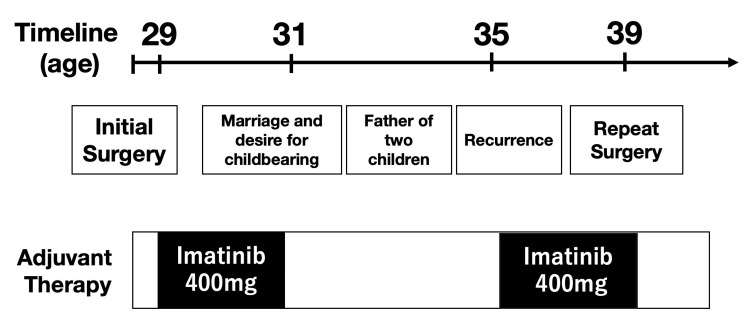
Timeline of clinical course Summary showing initial surgery at age 29, adjuvant imatinib therapy, treatment interruption for family planning leading to marriage and fatherhood of two children, tumor recurrence detection at age 35, reintroduction of imatinib with partial response, followed by surgical resection, and sustained disease control at age 39

Imatinib therapy was reinitiated postoperatively, and no evidence of disease recurrence has been observed during follow-up. The overall clinical course is summarized in Figure 7.

## Discussion

This case represents a rare and instructive clinical scenario involving spontaneous rupture of a giant gastric GIST in a young patient, successful emergency surgical management, subsequent disease recurrence following temporary treatment interruption for family planning, and durable disease control after repeat resection and reinitiation of imatinib therapy. The decade-long disease-free trajectory with two successful surgical interventions and a sustained response to imatinib underscores several important clinical and biological principles in GIST management.

Tumor rupture in GIST constitutes a surgical and oncologic emergency with profound prognostic implications. The SSG XVIII/AIO trial, the largest randomized study evaluating adjuvant imatinib duration, reported that patients with ruptured GIST had markedly inferior outcomes compared with those with non-ruptured disease, with 10-year recurrence-free survival rates of 21% versus 55% and overall survival rates of 59% versus 78%, respectively [8]. These findings corroborate earlier observations by Joensuu et al., demonstrating that tumor rupture carries prognostic weight comparable to, or exceeding, that of other high-risk features, including tumor size and mitotic count [9].

The definition of tumor rupture has been standardized by Nishida and colleagues, who proposed the Oslo criteria distinguishing major ruptures, including tumor spillage, fracture into the body cavity, blood-stained ascites, gastrointestinal perforation, macroscopic tumor invasion, and piecemeal resection, from minor defects that do not constitute true rupture [10]. In our case, the intraoperative finding of active bleeding and hemoperitoneum clearly indicated a major rupture, placing this patient in the highest-risk category regardless of other tumor characteristics.

The therapeutic success observed in this patient can be attributed in part to the molecular characteristics of the tumor. Approximately 75% to 80% of GISTs harbor activating mutations in the KIT gene, most commonly affecting exon 11, which encodes the juxtamembrane regulatory domain [2,3]. Heinrich et al. demonstrated that patients with KIT exon 11 mutations achieve partial response rates of approximately 84%, which are significantly superior to those observed in patients with exon 9 mutations (48%) or wild-type tumors (0%) [11]. Moreover, tumors with KIT exon 11 mutations exhibit prolonged event-free and overall survival compared with tumors of other genotypes.

A notable observation in our case is the preservation of imatinib sensitivity despite a three-year treatment-free interval and subsequent disease recurrence. The SSG XVIII/AIO trial long-term follow-up revealed that recurrent GISTs generally retain sensitivity to imatinib upon reintroduction, indicating that treatment interruption in the adjuvant setting does not necessarily select for resistant clones [12]. This observation is particularly relevant for patients with KIT exon 11 deletion/insertion mutations, who demonstrated a 10-year overall survival rate of 94% when treated with three years of adjuvant imatinib followed by appropriate salvage therapy upon recurrence [8]. The biological basis for this maintained sensitivity likely reflects the absence of significant selective pressure during the treatment-free interval, as clonal evolution toward resistance typically requires continuous drug exposure in the presence of residual disease.

The optimal duration of adjuvant imatinib in patients with ruptured GIST remains a subject of active investigation. Current guidelines recommend a minimum of three years of adjuvant therapy for high-risk patients based on the SSG XVIII/AIO trial, which demonstrated superior recurrence-free survival (71% versus 52% at five years) and overall survival (92% versus 85% at five years) with three years compared with one year of treatment [13,14]. However, given the extremely high recurrence risk associated with tumor rupture, extended or indefinite adjuvant therapy has been recommended by several expert groups [7].

The recently reported IMADGIST trial provides compelling evidence for extended adjuvant therapy, demonstrating that six years of imatinib significantly improved disease-free survival compared with three years (three-year disease-free survival (DFS): 87% versus 55%; hazard ratio (HR): 0.40; p = 0.008) in patients with high-risk GIST [15]. Notably, patients in the 35% to 70% recurrence risk stratum experienced the greatest benefit, with only 3% of those receiving six years of therapy experiencing recurrence compared with 39% in the three-year arm. These findings suggest that for young patients with ruptured GIST, even longer durations of adjuvant therapy may be warranted to optimize long-term outcomes.

The decision to perform repeat surgical resection for recurrent GIST must be individualized based on disease extent, response to systemic therapy, and surgical feasibility. Park et al. demonstrated in a propensity score-matched analysis that surgical resection of residual lesions after disease control with imatinib significantly improved progression-free survival (87.7 versus 42.8 months; p = 0.001) and overall survival compared with imatinib alone in patients with recurrent or metastatic GIST [16]. Complete (R0) resection is associated with superior outcomes, emphasizing the importance of careful patient selection and surgical planning.

In our patient, the identification of two discrete peritoneal recurrences that were amenable to complete resection provided an opportunity for combined modality treatment, which may have contributed to the favorable outcome. The ESMO-EURACAN-GENTURIS guidelines recommend consideration of surgery in patients with localized recurrence who have demonstrated stable disease or partial response to TKI therapy [7]. The key principle is that surgery should complement, rather than replace, systemic therapy in the management of recurrent GIST. A particularly challenging aspect of this case involved balancing oncologic risk with the patient's reproductive goals. The effects of imatinib on male fertility remain incompletely understood. Preclinical studies have demonstrated that imatinib inhibits c-KIT and PDGFR signaling pathways, which play critical roles in spermatogonial stem cell survival and Leydig cell function [17]. Human studies have yielded conflicting results, with some reports documenting decreased sperm parameters and testosterone levels during treatment, while others report no significant long-term impact on fertility after drug discontinuation [18,19].

Clinical evidence suggests that successful fatherhood is achievable in male patients receiving imatinib, with a recent multicenter study reporting that approximately 98% of male CML patients on TKI therapy did not experience adverse effects on fatherhood or offspring health [20]. Nevertheless, the potential for reversible gonadotoxicity supports considering sperm cryopreservation before initiating long-term TKI therapy in young male patients [18]. In our case, the decision to interrupt therapy for family planning was made through shared decision-making after thorough counseling regarding the substantial risk of recurrence. The subsequent disease recurrence, occurring three years after treatment discontinuation, validates these concerns while simultaneously demonstrating that effective salvage therapy remained possible.

Several limitations of this report warrant acknowledgment. First, mutational analysis of the primary and recurrent tumors was not performed, precluding definitive correlation between genotype and treatment response. Given the strong predictive value of KIT mutation status for imatinib sensitivity, molecular profiling should be considered a standard practice to guide treatment decisions [11]. Second, the duration of adjuvant therapy before interruption (two years) was shorter than the currently recommended three-year minimum, which may have contributed to the relatively early recurrence. Third, this single-case experience cannot address the question of whether alternative strategies, such as sperm cryopreservation followed by uninterrupted imatinib therapy, might have achieved superior oncologic outcomes while preserving reproductive options.

Future research directions should include prospective evaluation of extended (>6 years) adjuvant imatinib duration specifically in patients with ruptured GIST, the development of reliable biomarkers for predicting recurrence risk after treatment discontinuation, and systematic investigation of fertility preservation strategies in young GIST patients requiring long-term TKI therapy. The ongoing SSG XXII trial comparing three versus five years of adjuvant imatinib is expected to provide additional insights into optimal treatment duration [7].

## Conclusions

This report demonstrates the successful long-term management of a ruptured giant gastric GIST through coordinated emergency surgical intervention, adjuvant imatinib therapy, and salvage surgery for recurrent disease. The preservation of imatinib sensitivity despite treatment interruption allowed durable disease control following repeat resection and therapy reintroduction. This experience highlights the pivotal role of imatinib in both adjuvant and recurrent settings, the potential benefit of surgical intervention for localized recurrence, and the complex balance between oncologic risk and quality-of-life considerations in young patients with high-risk GISTs. Comprehensive patient counseling, individualized treatment planning with attention to molecular characteristics, and vigilant long-term surveillance are essential components of optimal care for this challenging patient population.
